# Air Nozzle Injury: Barotrauma Resulted From an Industrial Accident

**DOI:** 10.7759/cureus.61096

**Published:** 2024-05-26

**Authors:** Ashok N Mhaske, Nishi Gupta, Abhishek Mishra, Shubham Jaiswal, Chirag Dausage, Jyoti Meena, Gourav Goyal

**Affiliations:** 1 Department of General Surgery, People's College of Medical Sciences and Research Centre, People's University, Bhopal, IND

**Keywords:** surgical management, exploratory laparotomy, colon perforation, industrial accident, compressed air, colonic barotrauma

## Abstract

Industrial accidents involving compressed air can lead to significant colonic injuries, ranging from minor tears to complete perforations. This study investigates a case of colonic barotrauma in a 40-year-old male oil refinery worker who suffered symptoms of lower abdominal discomfort, distension, and tenderness following the application of compressed air to his anus. Diagnostic tests, including blood count, abdominal X-ray, and ultrasonography, indicated fecal impaction, dilated bowel loops, and free gas under the diaphragm. An exploratory laparotomy revealed a 4 cm x 2 cm hole in the colon at the hepatic flexure. There were also small breaks in the mucosa at the junction of the recto-sigmoid. We surgically repaired the perforation with primary closure, metrogyl lavage, and the placement of an intra-abdominal pelvic drain. Two weeks later, the patient recovered without any complications and was discharged. This case report highlights the severe risks of non-medical compressed air exposure, as well as the critical need for immediate surgical intervention and preventive safety measures in industrial settings.

## Introduction

The first documented case of barotrauma dates back to 1904, marking its origins in the 19th century. This incident marked the first occurrence of a fatal bowel rupture resulting from the introduction of compressed air through the rectum via a manually operated machine. The treating surgeon faces a challenge in identifying and managing these patients due to the unique nature of the injury and the scarcity of similar cases [1]. The use of high-pressure compressed air in industrial settings brings about significant risks if not handled properly. Pneumatic injuries caused by the improper use of compressed air can range from minor cuts and bruises to more severe injuries. In industrial settings that use high-pressure compressed air, accidental insufflation leading to colon injury is indeed a serious concern [2]. There is often a lack of awareness among the general public regarding the potential dangers of compressed air and its ability to penetrate barriers such as clothing and the anal sphincter, leading to severe colon injuries. This lack of awareness can contribute to accidents occurring, whether through careless use or, unfortunately, through practical jokes or pranks gone wrong.

## Case presentation

**Figure 1:**
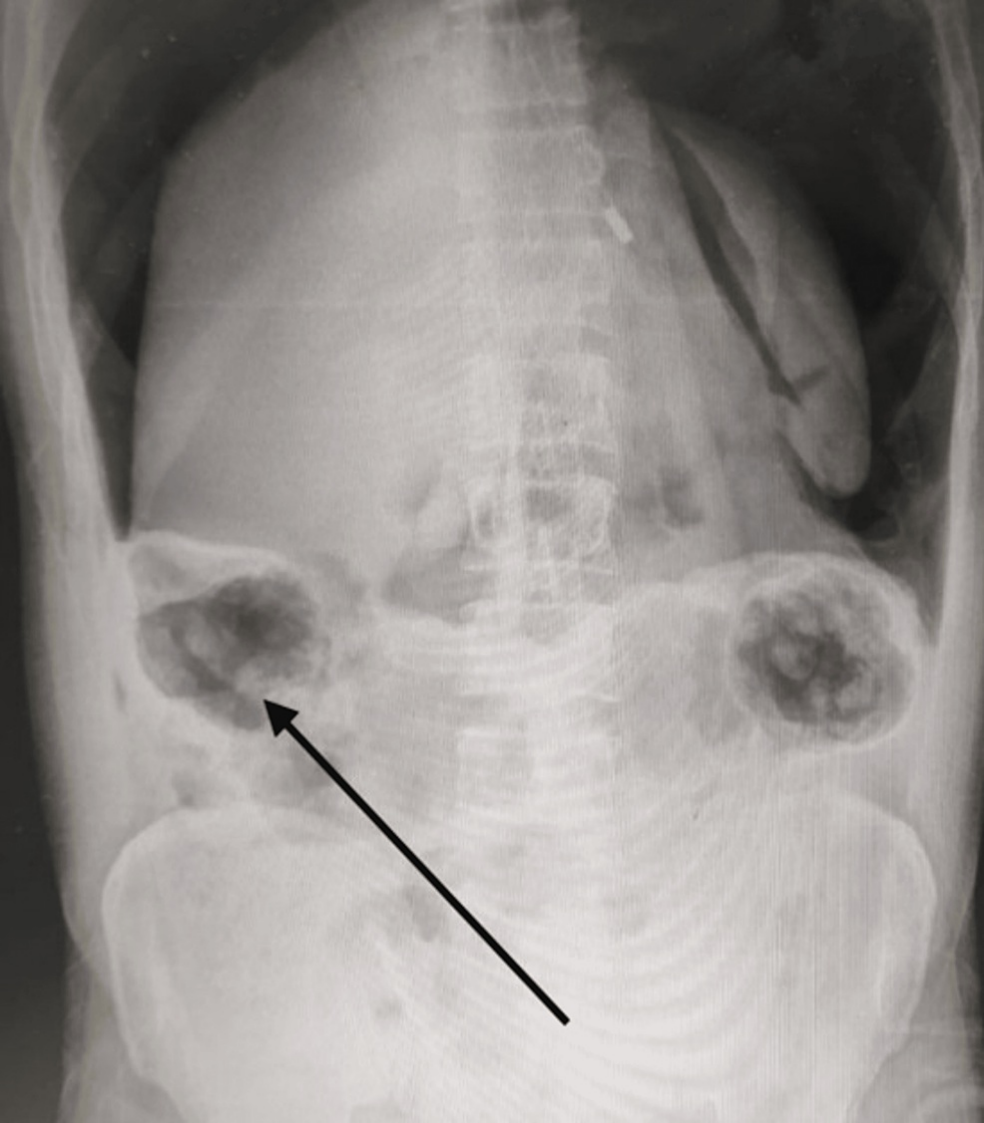
Abdominal X-ray of the patient with gas under the diaphragm suggestive of perforation peritonitis.

A 40-year-old male industrial worker, engaged in oil refinery projects, was hospitalized in the emergency department with lower abdominal discomfort persisting for three days. According to the patient's account, they experienced a blast of compressed air directed at their anus while they were working, initiated by their colleague. The patient came to the emergency room with complaints of pain in the abdomen and one episode of vomiting. The patient was vitally stable. On examination, the patient has abdominal distension with generalized tenderness and sluggish bowel sounds. No guarding or rigidity was present. On a digital rectal examination, fecal impaction was noted. During investigations, complete blood counts suggest high total counts with other normal parameters. The erect X-ray abdomen suggested a visible dilated bowel loop and free gas under the diaphragm; the outside ultrasonography (USG) scan revealed a mild to moderate amount of fluid in the inter-bowel space (Figure 1). We placed the patient on nil-by-mouth (NBM) status and inserted both Ryle's tube and a Foley catheter. Following initial resuscitation, the patient was scheduled for an exploratory laparotomy.

**Figure 2:**
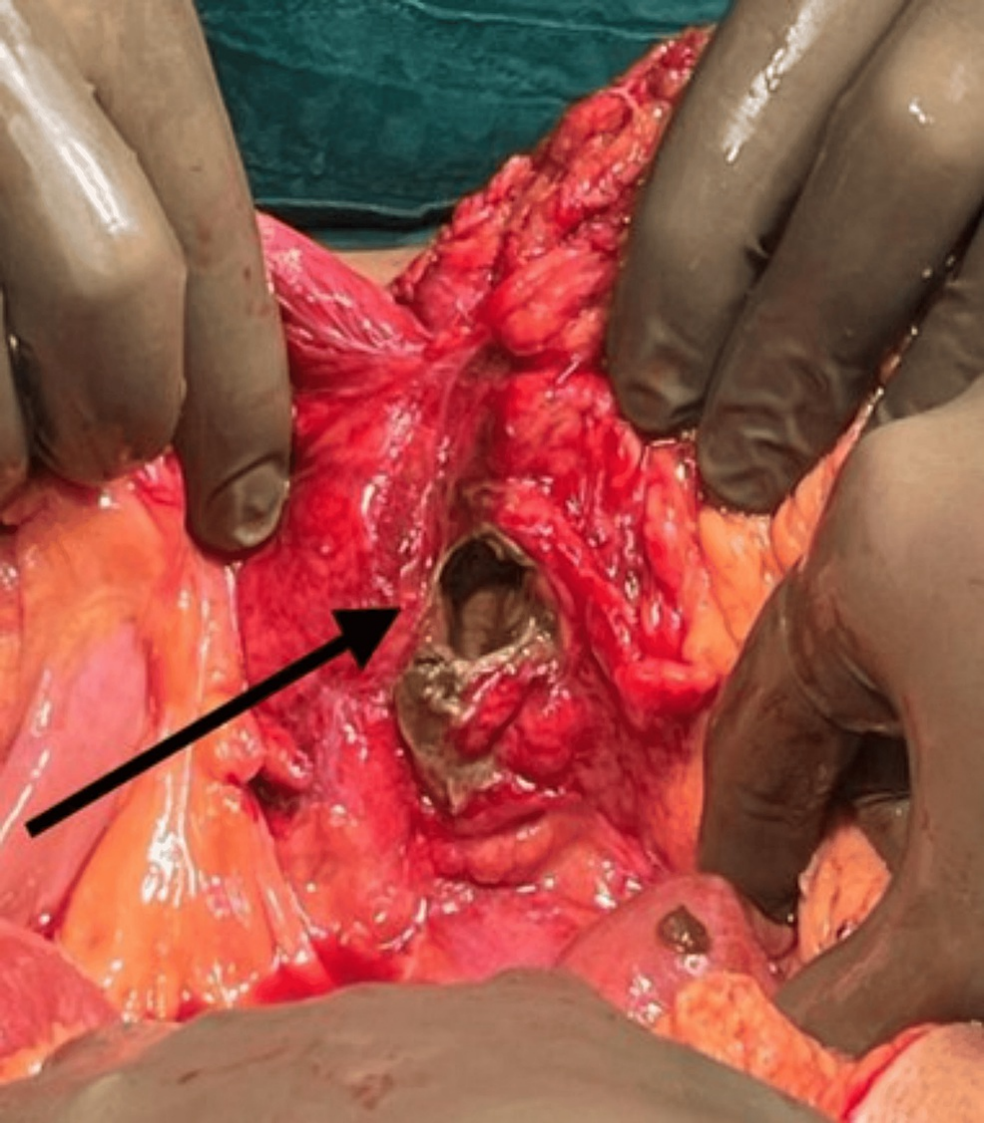
Surgical image showing the site of colonic perforation at hepatic flexure.

Figure 2 shows a colonic perforation measuring about 4 cm x 2 cm in the colon's hepatic flexure, along with multiple linear erythematous superficial breaks in the mucosa in the recto-sigmoid region. After freshening the perforated margins with 2-0 Vicryl and metrogyl lavage, we completed the primary closure of the perforation. An intra-abdominal pelvic drain was placed. The abdomen was closed in layers. The postoperative period went smoothly, with the removal of the drain on postoperative day (POD) eight. We discharged the patient after two weeks.

## Discussion

Barotrauma is defined as physical damage to body tissues caused by a difference in air pressure between the viscous material and its surroundings [1]. Barotrauma in the gut occurs when the pressure from either air or liquid surpasses the gut's capacity. Most cases of iatrogenic colonic barotrauma occur during a colonoscopy procedure. Reports indicate that their incidence ranges from 0.1% to 0.5% [1]. Colon injuries may happen anywhere along its length, ranging from minor tears in the outer layer to complete perforations through all layers. However, the recto-sigmoid junction and sigmoid colon are the most prevalent sites of injury in such cases [2]. Despite the growing prevalence of compressed air usage in contemporary society and occasional reports of non-medically induced colorectal injuries, fortunately, such injuries from compressed air remain infrequent. The industry typically uses jets with pressures of 50-100 pounds or higher. One can use the rubber hose without a nozzle; the nozzles are pipes with a diameter of one-quarter to one inch. Normally, one thinks of these jets in terms of experience with visible streams of water from a nozzle; however, the gaseous jet differs in that it is elastic and expands in all directions, adjusting to its environment, bending and twisting, and creating eddy currents [3,4]. Understanding the complexities of barotrauma due to compressed air exposure requires an integrated approach that considers both physiological principles and the impact of mechanical forces on the human body. Laplace's law, which states that the wall tension in the colon is directly proportional to the intramural pressure and colon diameter, provides a fundamental understanding of the forces at play [1]. Adding to this, the bowel wall's elasticity, notably in the mucosa - the most elastic and strongest layer - plays a critical role in how the colon can tolerate increased pressures [1,5]. From a mechanical perspective, Zunzunegui et al. have demonstrated that air pressures ranging from 3.5 to 8.8 kg/cm^2^ can force the anal sphincter open, essentially acting like a solid column [4]. Various factors, such as the speed of airflow, duration of exposure, and the bowel's ability to expand, influence the extent of injuries from such incidents [3]. The descending order of anatomical resistance to intraluminal pressure in different parts of the gastrointestinal (GI) tract, from the rectum to the stomach, makes it even harder to figure out where and how bad an injury could be [6].

The critical thresholds for bowel damage have been quantified in medical studies: the pressure required to cause colon perforation is greater than 0.109 kg/cm^2^ [7], and the pressure needed to induce a full-thickness tear is approximately 0.29 kg/cm^2^ [6]. Moreover, the velocity of airflow is as crucial as the pressure itself, especially at the recto-sigmoid junction. This area's limited mobility due to its bilateral fixation makes it particularly vulnerable to high-velocity air insufflation, leading to potential barotrauma [8-11]. In practical scenarios, such as those commonly found in industrial settings, compressed air can unexpectedly enter the bowel and cause barotrauma [12]. This type of injury is facilitated by the funnel shape of the buttocks and anus, which can inadvertently direct high-pressure air into the rectum [13]. Case studies have highlighted that non-iatrogenic barotrauma typically involves young male industrial workers who, due to ignorance or misuse, abuse compressed air equipment, often leading to severe injuries under prankish or perverse circumstances [14]. In medical settings, iatrogenic barotrauma is a risk during procedures such as colonoscopies. The perforation risk varies from 0.03% to 0.8% for diagnostic procedures and from 0.15% to 3% for therapeutic interventions [14]. Understanding both the physiological and mechanical factors involved in barotrauma is essential for preventing these injuries, whether they occur in industrial accidents or during medical procedures.

Colonic injuries resulting from barotrauma can range from subserosal tears to more severe full-thickness tears, which can compress the vena cava, decrease venous return to the heart, and potentially lead to hypotension and circulatory collapse [5]. These injuries often present as abdominal pain and distension, and if a colon perforation occurs, signs of peritoneal irritation can quickly develop. Additional complications may include respiratory distress due to reduced diaphragmatic movement from the pneumoperitoneum and respiratory alkalosis caused by hyperventilation from abdominal compartment syndrome. High intra-abdominal pressures can also lead to air migration, causing pneumomediastinum, pneumothorax, and extensive subcutaneous emphysema [7]. Diagnosis of these conditions often begins with an erect abdominal X-ray to detect pneumoperitoneum, followed by computed tomography (CT) of the abdomen, which is the most effective non-invasive method to confirm the severity and specific location of the injury [2]. Management strategies for colonic barotrauma focus primarily on addressing tension in the pneumoperitoneum and specific types of colonic injuries, ranging from conservative management to aggressive surgical intervention. Initial management involves resuscitation and stabilization of the patient, but surgical exploration is typically the mainstay of treatment. During surgery, injuries identified can range from serosal, muscular, to mucosal tears and even full-thickness perforations. The choice of surgical procedure - whether primary repair, Graham’s patch, decompression of the bowel, diversion colostomy or ileostomy with primary repair, or resection anastomosis - depends largely on intra-operative findings [8-10].

Specifically, management of colonic injuries may include conservative treatment or primary repair if the injuries are less severe. In cases of multiple tears, friable bowel, or severe fecal contamination, more extensive procedures such as resection with proximal enterostomy might be necessary [14]. The length of the perforation, the number of subserosal tears, and the degree of fecal contamination influence these decisions. In a noted case where there was minimal fecal contamination and subserosal tears were localized to the recto-sigmoid junction, primary closure was successfully performed. This integrated approach ensures that the treatment is tailored to the severity and specifics of the injury, optimizing patient outcomes in these critical situations [14].

## Conclusions

Operative protocol, though not standardized, remains an exploration in the case of perforation and conservation for non-perforated bowel injuries. The decision about ileostomy remains unclear, depending on the state of colonic perforation, local contamination, and presentation duration. To mitigate these risks, proper training and education on the safe handling and use of compressed air equipment are critical. Additionally, implementing strict protocols and guidelines for the use of compressed air, as well as regular equipment maintenance and inspections, can help prevent accidents and injuries in the workplace.
